# Spectral Domain Optical Coherence Tomographic Findings of Bietti Crystalline Dystrophy

**DOI:** 10.1155/2014/739271

**Published:** 2014-11-19

**Authors:** Ali Osman Saatci, Hasan Can Doruk, Aylin Yaman, Ferit Hakan Öner

**Affiliations:** Department of Ophthalmology, Dokuz Eylul University, Mithatpasa Caddesi, 35300 Izmir, Turkey

## Abstract

We analyzed the OCT features of 24 eyes of 12 patients with Bietti crystalline dystrophy (BCD) with the Heidelberg HRA2-OCT. Seventeen of 24 eyes were in intermediate stage of the disease and seven in advanced stage of the disease at the time of latest OCT examination performed in 2014. Outer retinal tubulations and retinal hyperreflective dots were present in 20 of 24 eyes. The remaining four eyes had advanced disease with very thin retina. Appearance of bright plaque on top of RPE-Bruch membrane was present in all eyes. Choroidal hyperreflective spots were noted in 19 of 24 eyes. The remaining five eyes had advanced disease stage with very thin choroid. Mean central macular thickness was 163.08 *μ*m ± 62.52 for all eyes (170.35 *μ*m ± 56.46 in eyes with intermediate disease and 145.42 *μ*m ± 77.2 in eyes with advanced disease). Mean subfoveal choroidal thickness was 95.37 *μ*m ± 55.93 for the study eyes (116.47 ± 46.92 *μ*m in eyes with intermediate disease and 44.14 *μ*m ± 42.43 in eyes with advanced disease). Choroidal hyperreflective spots were noted in 21 of 24 eyes (87.5%). SD-OCT shows the disease progression in retinal and choroidal layers delicately in eyes with BCD and expands our knowledge about the ongoing disease process.

## 1. Introduction

In 1937, Bietti [1] described three cases characterized by glistening, yellow-white intraretinal crystals in the posterior pole, atrophy of the retinal pigment epithelium, choroidal sclerosis, crystals in the paralimbal cornea, and onset in the third decade of life.

Now, it is well established that Bietti crystalline dystrophy (BCD) is caused by mutations of the CYP4V2 gene [2]. The intraretinal crystals that are hallmark of the disease disappear within time and are replaced by areas of chorioretinal atrophy associated with progressive visual deterioration [3–6].

Though the classical characteristics of BCD are well described, imaging modalities such as SD-OCT increase our understanding of the disease process and add new insight into anatomical location and clinical implications of chorioretinal crystals. We hereby describe the SD-OCT features of 24 eyes of 12 patients with BCD and discuss the relevant clinical correlations.

## 2. Subject and Methods

We retrospectively analyzed the data of 17 patients with BCD examined between 1995 and 2014 by one of us (AOS). The diagnosis of BCD was made on the basis of clinical evaluation after excluding other causes of crystalline retinopathy. After recalling the patients in March 2014, we could reexamine 12 of them and reperform the OCT analysis. Those 12 patients (24 eyes) comprised the study group.

At the time of latest OCT examination in 2014, patients were classified into three disease stages as proposed before [7–9].

- Early disease: crystals are scattered throughout the posterior pole and midperiphery with chorioretinal atrophy limited to posterior pole.
- Intermediate disease: crystals are very few or absent at the posterior pole but still visible outside the area of atrophy up to the equator. Atrophy extends beyond the macula.
- Advanced disease: there is almost complete chorioretinal atrophy with occasional crystals.

Heidelberg HRA2-OCT (Heidelberg retina angiograph-optical coherence tomography, Heidelberg Engineering, Heidelberg, Germany) was the SD-OCT used throughout the study. Analyzed OCT parameters were stated below: volume analysis was selected to scrutinize retinal layers (number of horizontal B-scans: 23, pattern size 20° × 18°, and the distance between B-scans: 247 *μ*m) for the detection of intraretinal hyperreflective dots, bright plaques on top of RPE-Bruch membrane complex, and outer retinal tubulations (ORTs). Central macular thickness was measured. EDI-OCT was also performed to look for choroidal hyperreflective spots and measure the subfoveal choroidal thickness. Choroidal thickness was measured manually from the inner border of the sclera to the outer border of the RPE vertically using the calipers of the Heidelberg reader subfoveally and at 500 *μ*m intervals for 2.5 mm nasal and temporal to the center of the fovea as described by Margolis and Spaide [11]. To make a comparison, central macular thickness and subfoveal choroidal thickness were measured in a healthy age-matched population comprised of 31 subjects (mean age, 45.77 ± 13.33 years).

## 3. Results

Nine patients were male and three female. The mean age of the group in 2014 was 45 years (range, 24–66 years). Patients' characteristics and OCT features are summarized in Table 1.

Seventeen of 24 eyes were in intermediate stage of disease and seven in advanced disease stage. Mean central macular thickness was 163.8 *μ*m ± 62.52 for all study eyes. However it was 170.35 *μ*m ± 56.46 for the eyes with intermediate disease stage and 145.42 *μ*m ± 77.2 for the eyes with advanced disease stage. Mean central macular thickness was 227.45 ± 12.40 *μ*m (range, 205–262 *μ*m) for the control eyes. Mean subfoveal choroidal thickness was 95.37 *μ*m ± 55.93 for all study eyes. It was 116.47 *μ*m ± 46.92 for the eyes with intermediate disease stage and 44.14 *μ*m ± 42.43 for the eyes with advanced disease stage. Mean subfoveal choroidal thickness was 252.59 *μ*m ± 31.41 *μ*m (range, 189–306 *μ*m) for the control eyes.

**Figure 1:**
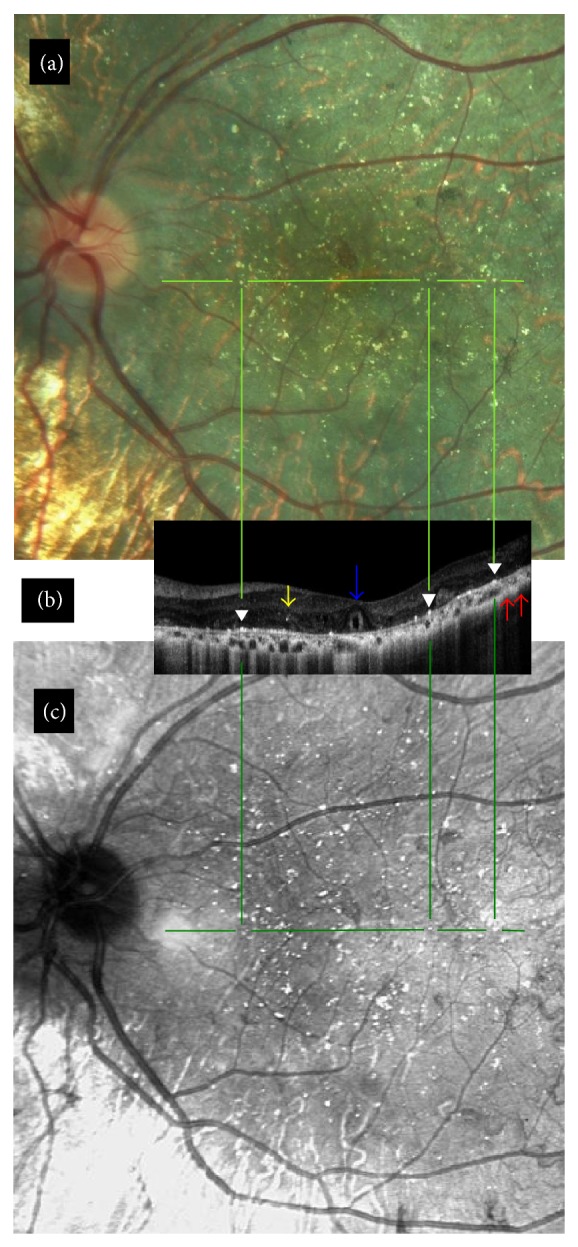
Case 3, left eye. (b) Transvers SD-OCT section. Blue arrow: outer retinal tubulations alone or with high reflective spots within. White arrowheads: bright reflective spots on top of RPE-Bruch membrane complex. Yellow arrow: intraretinal hyperreflective dot. Red arrows: choroidal hyperreflective spots. (a) and (c) Color fundus and reflectance images and the relationship between bright reflective spots on top of RPE-Bruch membrane complex and crystals.

**Figure 2:**
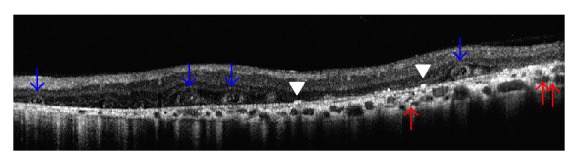
Case 2, left eye. Transvers SD-OCT section. Blue arrows: outer retinal tubulations alone or with high reflective spots within. White arrowheads: bright reflective spots on top of RPE-Bruch membrane complex. Red arrows: choroidal hyperreflective spots.

**Figure 3:**
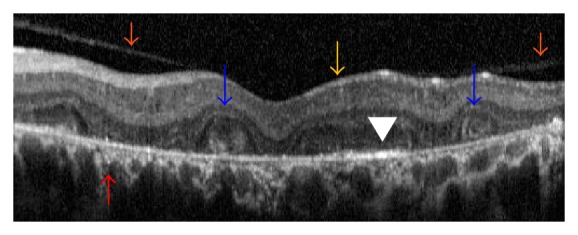
Case 1, left eye. Transverse OCT section showing the associated vitreomacular adhesion (orange arrows) besides outer retinal tubulations (blue arrows), choroidal hyperreflective spots (red arrow), and bright reflective spots on top of RPE-Bruch membrane complex (white arrowhead).

While ORTs were present in all 17 eyes with intermediate stage, they were present in only three of 7 eyes with advanced disease. Similarly intraretinal hyperreflective dots were present in all 17 eyes with intermediate disease and three of seven eyes with advanced disease. However, bright plaques on top of Bruch membrane-RPE complex were present in every eye at least in one OCT section. Choroidal hyperreflective dots were present in 19 of 24 eyes. The remaining five eyes had advanced disease stage. Vitreomacular abnormalities, such as vitreomacular adhesion and vitreomacular traction, were noted in six eyes. OCT findings of two eyes of two cases were seen in Figure 1 (together with color fundus and reflectance images) and Figure 2.  Figure 3 shows the OCT section of a case with vitreomacular adhesion in addition to other findings.

## 4. Discussion

Advances in OCT technology add new dimensions to morphologic characteristics of many retinal diseases. Though the clinical features of BCD are well known SD-OCT reveals additional morphologic findings. Some of the previously reported macular changes are subretinal neovascularization [12–15], macular hole [16], cystoid macular edema [10, 17], and subfoveal sensorial retinal detachment [18].

Main OCT features in BCD are outer retinal tubulations (ORTs) and hyperreflective lesions localized almost in all retinal layers [19–27]. ORT is usually located in the outer nuclear layer of the retina and appears as round or ovoid hyporeflective spaces with hyperreflective borders in the B-scan SD-OCT sections [23]. ORTs seem to represent rearrangement of the photoreceptor layer in response to severe and rapid retinal degeneration of photoreceptors. ORTs are somehow relatively more commonly described in BCD when compared to other retinal dystrophies such as retinitis pigmentosa and cone dystrophy [22, 23]. Kojima et al. [22] analyzed the SD-OCT data of a group of patients with various types of dystrophies and demonstrated that ORTs were present in 10 of 292 retinitis pigmentosa patients (3.4%), two of 16 cone dystrophy patients (12.5%), and all the 12 patients with BCD (100%). Iriyama et al. [23] investigated 112 patients with RP, 58 with cone dystrophy, and 7 with BCD and ORTs were present in 0 of 112 patients with RP, unilaterally in 3 of 58 patients with cone dystrophy, and bilaterally in 5 of 7 patients with BCD. In the previous two studies, the relationship between ORT and disease severity was not elaborated in BCD. In our group ORTs were present in all stage 2 eyes and 3 of 7 eyes with advanced disease stage. Perhaps in eyes with severe retinal thinning, ORTs could not be observed due to already established extensive degeneration. Contrary to reports summarized above, only 3 eyes out of 22 eyes (13.6%) with BCD had ORTs when examined with Cirrus OCT in a very recent study from a Chinese population [26].

A myriad of bright reflective spots of various configurations is present in SD-OCT sections of patients with BCD [18]. Hyperreflective retinal dots can be classified into three types: (1) highly reflective spots in the inner retina, (2) bright reflective plaques on top of the Bruch membrane, and (3) partially encapsulated reflective plaques. However only some of the hyperreflective dots seem to correspond to the crystalline deposits [19, 21]. Many authors argued that hyperreflective spots located in or on the RPE-Bruch membrane complex corresponded to the crystals [21, 25]. The rest of the hyperreflective dots may be related to inflammatory cells, protein deposits, a glial response to retinal degeneration, or even artefacts [25]. In a very recent study, Gocho et al. [27] stated that the crystals noted in the infrared images were found adjacent to relatively healthy RPE and where the outer nuclear layer was preserved as shown with Cirrus OCT. In our study, inner retinal hyperreflective dots were present in 20 of 24 eyes and only in four eyes with advanced disease there were none. On the other hand bright plaque was shown in all of the study eyes. The presence of hyperreflective spots suggestive of crystals in choroid is even more controversial. Only in some reports, choroidal hyperreflective dots attributed to crystals in the choroid were described [24]. There seem to be two possible explanations for the presence of choroidal crystals. First, there are really an increased number of crystals in the choroid under the atrophic retina or alternatively SD-OCT allows the cell reflectivity beneath the retinal atrophy to be distinguished more precisely. In our group of eyes, choroidal hyperreflective dots were present in 20 of 24 eyes. Only in four eyes with advanced stage of disease with severe chorioretinal thinning, there were no choroidal hyperreflective dots.

In our healthy control group, the mean central macular thickness and mean subfoveal choroidal thickness were 227.45 *μ*m and 252.59 *μ*m, respectively. Comparatively, mean central macular thickness and mean subfoveal choroidal thickness were 170.35 *μ*m and 116.47 *μ*m, respectively, for the eyes with intermediate disease stage and 145.42 *μ*m and 44.14 *μ*m for the eyes with advanced disease stage, respectively. Chorioretinal thinning seems to be in correlation with disease severity.

Just very recently, Halford et al. [25] reviewed the data of 20 patients from 17 families recruited from a multicenter British population over a period of 13 years. On clinical examination, all patients had retinal crystals at the posterior pole with varying degrees of RPE and inner choroidal atrophy. Outer retinal tubulations were present in all patients except a single case with an early stage of disease. They argued that crystals were generally situated on or in the RPE/Bruch's complex but could disappear over time with associated RPE disruption.

In light of our observations, we believe that SD-OCT shows the disease progression in retinal and choroidal layers very delicately and enables us to judge the disease severity.

## Figures and Tables

**Table 1:** Patients' characteristics and OCT findings.

Patient number	Age	Gender	Eye	Stage	CMT (*μ*m)	SCT (*μ*m)	ORT	Intraretinal hyperreflective dots	Bright plaque	Choroidal hyperreflective dots	Vitreomacular interface status
1	34	M	OD OU	2	203	125	+	+	+	+	VMA
				2	153	131	+	+	+	+	VMA
2	45	M	OD OU	3	141	140	+	+	+	+	—
				2	181	108	+	+	+	+	—
3	33	F	OD OU	2	194	140	+	+	+	+	—
				2	198	104	+	+	+	+	—
4	66	M	OD OU	2	225	95	+	+	+	+	—
				2	100	52	+	+	+	+	—
5	66	F	OD OU	2	41	69	+	+	+	+	—
				2	58	39	+	+	+	+	—
6	28	M	OD OU	2	245	210	+	+	+	+	—
				2	227	179	+	+	+	+	—
7	65	M	OD OU	3	161	22	−	−	+	−	VMT
				3	160	30	−	−	+	−	VMT
8	24	M	OD OU	3	179	24	−	−	+	−	—
				3	274	32	−	−	+	−	—
9	45	F	OD OU	2	160	181	+	+	+	+	VMA
				2	198	153	+	+	+	+	VMA
10	39	M	OD OU	3	56	29	+	+	+	+	—
				3	47	31	+	+	+	−	—
11	56	M	OD OU	2	183	120	+	+	+	+	—
				2	183	116	+	+	+	+	—
12	44	M	OD OU	2	152	66	+	+	+	+	—
				2	195	92	+	+	+	+	—

CMT: central macular thickness, SCT: subfoveal choroidal thickness, VMA: vitreomacular adhesion, VMT: vitreomacular traction, and ORT: outer retinal tabulation.

^*^Patient number 1 had also bilateral cystoid macular edema [10].
